# Long-Term Outcomes of Definitive Chemoradiotherapy for Early-Stage Extranodal Natural Killer/T-cell Lymphoma, Nasal Type: A Retrospective Analysis From a Single Center

**DOI:** 10.7759/cureus.34348

**Published:** 2023-01-29

**Authors:** Atsuto Katano, Hideomi Yamashita

**Affiliations:** 1 Radiology, University of Tokyo Hospital, Tokyo, JPN

**Keywords:** nk cell lymphoma, retrospective research, radiotherapy (rt), t-cell lymphoma, nasal cavities

## Abstract

Background

Nasal extranodal natural killer (NK)/T-cell lymphoma (ENKTL) is a rare type of lymphoma with characteristic histological features. Although radiotherapy can achieve a high response rate, long-term efficacy and safety are yet to be established.

Methodology

Using electronic health records, we identified relevant patients treated at our hospital from August 2005 to August 2015. We enrolled patients with pathologically confirmed ENKTL treated with curative intent radiotherapy.

Results

We included 13 patients who underwent definitive radiotherapy in the analysis, comprising 11 males and 2 females and a median age of 53 years (range: 28-73). The median follow-up period was 113.4 months. The overall survival at 5 and 10 years was 92.3% (95% confidence interval [CI]: 57-99 %) and 68.4% (95% CI: 29-89 %), respectively. The most common radiation-related late-term toxicity was sinus disorder (Grade 1-2) in 11 patients (85%). Radiation-related grade 3 to 5 toxicities were not observed.

Conclusion

The present retrospective study elucidated the long-term safety and effectiveness of curative intent radiotherapy in patients with localized ENKTL.

## Introduction

Natural killer (NK) cells provide a rapid immune response against virus-infected cells and other intracellular pathogens. Mature NK cell neoplasms exhibit three subtypes: extranodal NK/T-cell lymphoma, nasal type (ENKTL), aggressive NK-cell leukemia, and chronic lymphoproliferative disorders of NK cells [1]. All these subtypes are rare diseases, and there is a serious paucity of evidence regarding treatment strategies.

Chemotherapy and radiotherapy are essential treatment modalities for patients with lymphomas. As initial therapy for localized ENKTL cases, patients who underwent radiotherapy experienced a significantly higher overall survival (OS) and progression-free survival (PFS) than chemotherapy-treated patients (5-year OS: 83.3% vs. 28.6%, p = 0.0269; 5-year PFS: 83.3% vs. 27.1%, p = 0.0247) [2]. However, radiotherapy alone is insufficient to achieve systemic disease control, and an effective combination of chemotherapy and radiation therapy is expected to improve survival rates [3]. Although several B cell type lymphomas have been found to favorably respond to the regimen comprising cyclophosphamide, adriamycin, vincristine, and prednisone (CHOP), ENKTL expresses P-glycoprotein, which is well-known to mediate multi-drug resistance (MDR); therefore, it is considered less responsive to CHOP therapy [4]. To overcome MDR, various chemotherapy regimens using radiation therapy have been proposed.

To the best of our knowledge, few studies have reported the long-term safety and feasibility of radical radiotherapy (RT). Herein, we report the long-term clinical outcomes of patients with ENKTL who were treated with radical chemoradiotherapy at our institution.

## Materials and methods

We identified patients who underwent treatment at our hospital from August 2005 to August 2015 using electronic health records, followed by a manual reviewal to assess eligibility. We included patients with pathologically confirmed ENKTL and treated with curative intent radiotherapy. Patients who underwent radiotherapy for palliative intent and those who could not accomplish the planned treatment sequence were excluded. This study was approved by the Institutional Human Research Ethical Committee. Clinical staging of all patients was determined by positron emission tomography-computed tomography according to the Lugano classification criteria [5].

**Figure 1:**
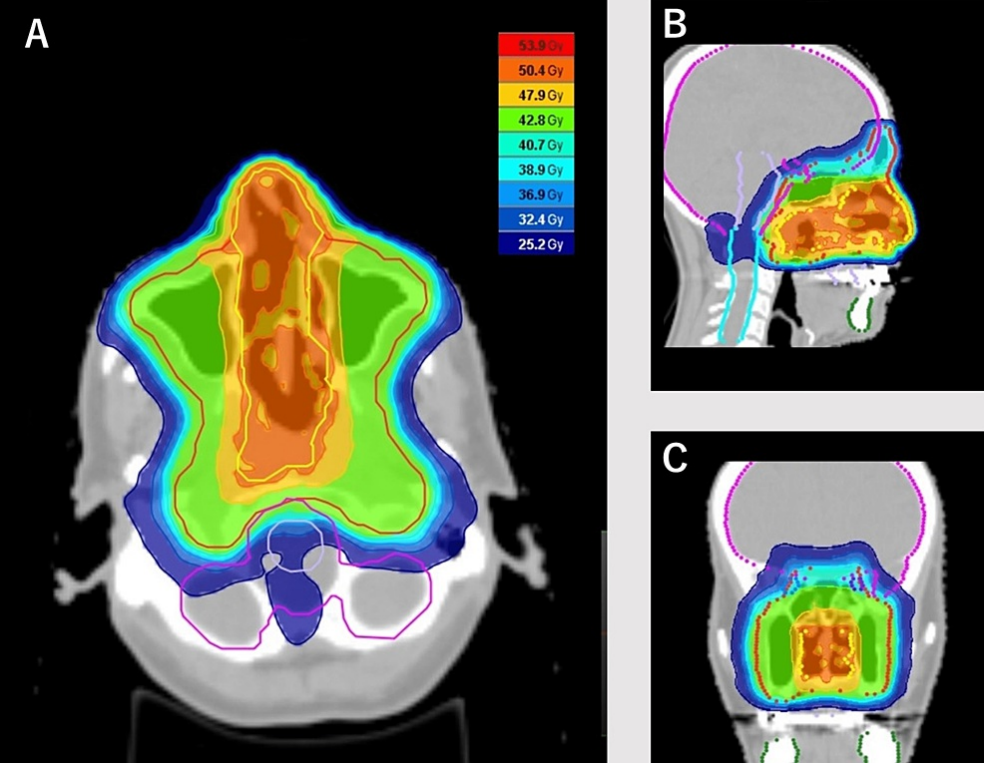
Radiation dose distribution Axial (A), sagittal (B), and coronal (C) images of treatment planning for a representative patient with stage I extranodal natural killer/T-cell lymphoma, nasal type.

The patients were treated with radical radiotherapy, with a total dose of 50-60 Gy in 25-30 fractions (1.8-2 Gy per fraction). The radiotherapy techniques utilized were three-dimensional conformal radiotherapy (3D-CRT) and intensity-modulated radiotherapy (IMRT) (Figure 1, Table 1).

**Table 1:** Dosimetric parameters of radiotherapy This table shows the dosimetric parameters of the patient displayed in Figure 1. PRV: Planning organ at risk volume, D_max _:Maximum point dose of the organ, D_1cc_:  Maximum dose delivered to a volume of 1 cc, D_mean _: Mean dose to the organ.

Organs at Risk	Parameter	Value
Spinal cord PRV	D_max_	43.62Gy
	D_1cc_	34.30Gy
Brain PRV	D_max_	47.45Gy
	D_1cc_	45.50Gy
Brainstem PRV	D_max_	40.26Gy
	D_1cc_	35.45Gy
Optic nerve PRV (Left / Right)	D_max_	42.35Gy / 42.17 Gy
Optic chiasm PRV	D_max_	41.47Gy
Eyeball (Left / Right)	D_max_	37.53Gy / 38.25 Gy
Lens (Left / Right)	D_mean_	4.95 Gy / 5.30 Gy
Parotid glands (Left / Right)	D_mean_	18.39 Gy / 15.04 Gy
Chochlea(Left / Right)	D_mean_	29.60 Gy / 31.25 Gy
Submandibular glands (Left / Right)	D_mean_	1.44 Gy / 1.48 Gy

The treating physician decided to administer concurrent systemic treatment. The regimen comprising dexamethasone, etoposide, ifosfamide, and carboplatin (DeVIC), was the most frequently employed concurrent regimen with radiotherapy.

Data analyses were performed using the R software package. Statistical significance was set at ˂0.05. PFS was calculated from the date of radiotherapy initiation until disease progression or death from any cause. OS was estimated from the date of radiotherapy initiation to death from any cause. Considering patients lost to follow-up, OS data were censored on the date the patient was last seen alive. Toxicity data were evaluated from electronic medical records and graded retrospectively based on the Common Terminology Criteria for Adverse Events v5.0.

## Results

Thirteen patients who were treated with radiotherapy were included in the present analysis (Table 2).

**Table 2:** Basic characteristics Basic characteristics of 13 patients who underwent curative radiation therapy for early-stage natural killer/T-cell lymphoma, nasal type. IMRT: Intensity Modulated Radiation Therapy

Variables		Number	Percentage
Age (Median)		53 (Range: 28-73)	
Gender	Male	11	85
	Female	2	15
Karnofsky Performance Status	100	1	8
	90	9	69
	80	3	23
Clinical stage	I	10	77
	II	3	23
Radiotherapy technique	3D-CRT	10	77
	IMRT	3	23
Dose and fractions	50 Gy in 25 fractions	6	46
	50.4 Gy in 28 fractions	3	23
	60 Gy in 30 fractions	4	31
Chemotherapy	CHOP	3	23
	DeVIC (2/3 dose)	10	77

Of these, 11 were males and 2 were females, with a median age of 53 years (Range:28-73). All patients completed radiation treatment with a total dose of 50.4-60 Gy in 25-30 fractions. Ten patients were classified as stage I and three as stage II. The median serum lactate dehydrogenase (LDH) level was 224 IU/L, ranging between 158-635 IU/L. According to the normal level (124-222 IU/L) established at our institution, seven patients were considered to exhibit elevated serum LDH levels. One patient received high-dose chemotherapy with autologous hematopoietic stem cell transplantation as consolidation therapy after definitive radiotherapy.

**Figure 2:**
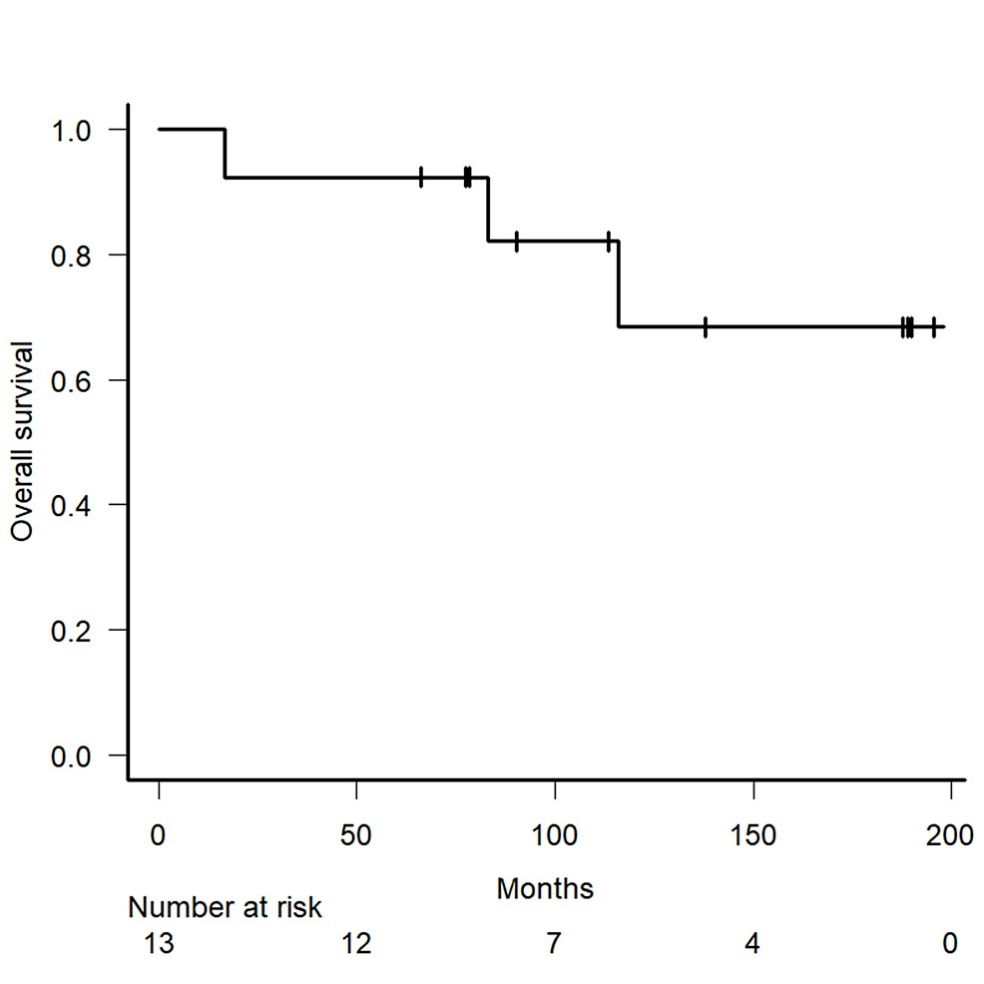
Kaplan–Meier plot Kaplan–Meier plot of the overall survival rate for the entire study cohort.

The total follow-up period ranged from 16.7 to 195.5 months. The median follow-up period was 113.4 months. The OS rates at 5 and 10 years were 92.3% (95 % confidence interval (CI): 57-99 %) and 68.4% (95% CI: 29-89 %), respectively (Figure 2). The PFS rates at 5 and 10 years were 84.6% (95 % CI: 51-96 %) and 76.9% (95% CI: 44 - 92 %), respectively.

Three patients (23.1%) developed recurrent disease; of these, two presented with a local recurrence and received the DeVIC regimen and unknown treatment (treated in another hospital) as salvage therapy. The third patient presented with recurrence in the oral cavity and was treated with a regimen comprising cyclophosphamide, vincristine, adriamycin, and dexamethasone (hyper-CVAD). Three deaths occurred during the observational period. Two patients had disease progression of the primary disease, and one died of rectal cancer, which was deemed to be unrelated to ENKTL treatment after 4 years of radiotherapy.

The most common radiation-related late-term toxicity was sinus disorder (Grade 1-2) in 11 patients (85%). Grade 2 middle ear inflammation was observed in 3 patients, grade 2 hearing impairment in 3 patients, grade 2 periodontal disease in 3 patients, and grade 2 cataract in 2 patients. Grade 3 and 4 toxicities were not observed.

## Discussion

The clinical outcomes of the present study were comparable to those previously reported. The Japan Clinical Oncology Group (JCOG) conducted a phase I/II trial (JCOG0211) to evaluate the effectiveness of concurrent chemoradiotherapy for localized ENKTL [6]. The concurrent chemotherapy regimen with the radiotherapy was a two-thirds dose of the DeVIC regimen, resulting in a 2-year OS rate of 78% (95% CI: 57 - 89%) with a median follow-up of 32 months. The Phase II trial published by Kim et al. showed the effectiveness of concurrent chemoradiotherapy with an adjuvant VIPD regimen comprising etoposide, ifosfamide, cisplatin, and dexamethasone. They reported that the 3-year OS and PFS rates of the cohort were 86% (95% CI: 74 - 98%) and 85% (95% CI: 72 - 98%), respectively [7]. Dong et al. reported that the 5-year OS and PFS rate for the ENKTL patient treated by sequential chemoradiotherapy is 89% and 82%, respectively [8]. This sequential chemotherapy regimen included dexamethasone, ifosfamide, cisplatin, and etoposide (DICE) combined with L-asparaginase, which is a characteristic agent that induces selective apoptosis of NK tumors [9]. The OS of the present study tended to be slightly better than these, which might be caused by the relatively high rate of stage I cases. The strength of our study was the relatively long-term follow-up the median period was approximately 10 years, which implies excellent long-term tumor control outcomes and safety were achieved by chemoradiotherapy.

There are some studies focusing on prognostic factors and biomarkers for risk stratification approaches in the treatment of ENKTL. Suzuki et al. identified the prognostic factors for ENKTL, which included non-nasal type, clinical stage, performance status, and extranodal invasion [10]. Lee et al. pointed out four prognostic factors: systemic symptoms, clinical stage, serum LDH level, and regional lymph node metastasis [11]. Interestingly, both these studies concluded that clinical stage was a prognostic factor, whereas age at diagnosis was not deemed a prognostic factor. Serum Epstein-Barr virus DNA levels, assessed using real-time quantitative polymerase chain reaction, could also be used for diagnosis and as indicators of disease progression [12].

Recently, novel approaches for the treatment of ENKTL were proposed, especially in targeting the tumor immune system. Pembrolizumab, an anti-programmed death 1 antibody working as an immune checkpoint inhibitor, provided an objective response rate of 100% in patients with ENKTL after failed initial treatment [13]. Additionally, Kim et al. analyzed soluble and exosomal programmed death-ligand 1 levels in serum samples and identified responders to pembrolizumab administration for NKTCL [14]. Sintilimab, another immune checkpoint inhibitor evaluated in a multicenter phase 2 trial conducted by Tao et al. also reported favorable efficacy and was well-tolerated with adverse events [15]. These novel therapies have the potential to improve the survival rate as salvage treatment in relapsed or refractory cases.

This study was limited by the relatively small number of patients confined to a single institution. Given the retrospective observational nature of the present study, the possibility of a selection bias cannot be excluded, which may have influenced our results. Further studies are needed to confirm the observations in the present study.

## Conclusions

This retrospective study elucidated the long-term safety and effectiveness of curative intent radiotherapy for patients with localized ENKTL. No radiation-related late adverse events graded as 3 or higher were observed in our cohort even though the patients treated by 3D-CRT were relatively dominant.
